# Hospital Meetings, &c.

**Published:** 1898-02-05

**Authors:** 


### HOSPITAL MEETINGS, &C.

### THE HOME HOSPITALS ASSOCIATION.

This association, founded by Sir Henry (then Mr.) Burdett in 1878, will attain its majority next year. Fitzroy House, Fitzroy Square, W., which is the name of the present hospital, was opened in 1880, and the result has proved, in spite of the numerous home hospitals which have sprung up in London and elsewhere since then, that the hospital, being well managed and rightly conducted, can still hold its own and take the first place amongst the home hospitals in England. Fitzroy House, unlike all other homes, is not conducted on commercial principles nor, on the other hand, is there anything of an eleemosynary character about it. It was established to provide accommodation for those who could afford to pay a reasonable sum for their lodging, nursing, and diet when ill; the object was not that of gain but the advantage of those who, being inadmissible at the general hospitals, yet have not the means to provide when ill the necessary comforts at their own homes. It is managed by a committee chosen from the governors and members only. This committee consists of 15 and meets once a month. There is also a medical board of reference, which is appointed by the committee for the better management of the professional business of the association in matters relating to the medical administration and hygiene of the home. Recently it has been found rather difficult to find a sufficient number of governors and members to sit on or attend the committee of management. It nominally at present consists of 15 members, but during the past year eight of this number, from one cause or another, found it impossible to attend any of the meetings. The result was a quorum was not always easily obtainable. To obviate this in the future, it was decided last December to call an extraordinary general meeting of the governors and members to consider the advisability of so altering article 15 of the articles of association as to permit gentlemen interested in this association, who were not already governors to sit on the committee. This meeting was held, after due notice, on the 25th ult., when it was unanimously agreed that article 15 should be amended as follows: "The said committee shall consist of 15 members, including the president and treasurer, who shall be *ex-officio* members of the association. Any governor, member, or other person interested in the association shall be qualified for election to the said committee." This resolution will be submitted for confirmation as a special resolution at an extraordinary general meeting to be held at Fitzroy House, W., on February 15th, at four p.m., immediately after the annual meeting.

### THE ITALIAN HOSPITAL.

The annual meeting of the Italian Hospital, held on Saturday at the Italian Embassy in Grosvenor Square, was of unusual interest, owing to the fact that plans of the new hospital to be erected on the site of the old building, which has been pulled down, were submitted to the governors. The chair was taken by Signor G. B. Ortelli, the founder of the institution, and among those present were: General Ferrero (Italian Ambassador), Sir G. Fayrer, Signor P. Micali, Dr. Naumann, Dr. Castaneda, Mr. R. E. Worsley, Mr. Francis Miller, and Signor Ferrari (secretary). According to the report read to the meeting, the total receipts for the year amounted to £1,726 17s., and the expenditure to £839 12s., leaving a balance of £887 5s. The donations had been considerably larger than usual, while the annual ball had yielded no less than £435 9s. In consequence of the demolition of the building the number of in-patients treated was only 122, against 253 in 1896. The out-patients numbered 4,617, against 5,158; and of these, 2,165 were Italians, and 2,043 English, the remainder consisting of members of various other nationalities. The report having been adopted, Signor Tomaso Sperati and Signor Cav. Righetti were added to the committee, and Mr. Cutler, the well-known architect, of Queen Square, then produced the plans of the new building and explained them to the meeting. The old hospital was in reality a dwelling-house, but the new building which is to take its place is to be a handsome edifice in the Italian style of architecture, and Mr. Cutler states that it will be equipped in accordance with the latest developments of sanitary science. The main entrance will be in Queen Square, but the out-patient department will be entered only from Devonshire Street. It is proposed that this department shall be quite distinct, but means of communication with the main building will be provided by private doors, to be used only by the staff. In addition to accommodation for the medical and surgical officers, there will be an accident ward and operating-room on the ground floor. On one side of the staircase on the first floor there will be a men's ward with fourteen beds, and on the other four single wards, and a ward containing four beds. The second floor will comprise a ward for women containing eight beds, a children's ward with five beds, two wards containing two beds each, a single ward, and an isolation ward. This last-named ward is cut off from the main building by a passage, open to the air at both ends. The sisters' dormitory, dining-room, and sitting-room, as well as a chapel, are to be located on the third floor, and the flat roof, which will be sheltered, will provide an exercise ground for the patients. It is proposed to construct the floors of teak or oak, the dados will be lined with glazed materials, and the wards will be heated with open fires and warm-air coils. The windows will be so arranged that the building can be air-swept as often as may be desired, and Mr. Cutler states that it will be possible to change the air in the wards three or four times an hour. The staircase, it may be said, will be shut off the wards on both sides by cross-ventilating lobbies. The building, which will cost about £15,000, is to be put in hand at once, and will, it is expected, be completed by the end of the year. Meanwhile, out-patients are treated at the temporary premises in Orange Street, Red Lion Square, where there is also accommodation for one or two in-patients in cases of extreme urgency.

### THE AFTER-CARE ASSOCIATION.

Sir William Broadbent very kindly permitted the annual meeting of the After-Care Association to be held at his house, in Brook Street, on January 31st, at three p.m., and took the chair himself. He made a few introductory remarks, heard the report, which was read by the secretary, and the speeches of Dr. Savage, the Archdeacon of Westminster, Dr. White, and the Rev. E. S. Hilliard, who respectively moved, seconded, and supported the adoption of the report, and was then compelled to leave as he had another engagement. The election of the officers and council was proposed by Mr. Deputy White and seconded by Dr. Norman Kerr, and carried unanimously, and the meeting closed with a vote of thanks to the Chairman and Lady Broadbent, which was proposed by Dr. Rayner and seconded by the Rev. Henry Hawkins, the originator of the association. Two facts were universally acknowledged by the speakers. One that the year ending December 31st, 1897, has been the most prosperous and useful in the history of the association, the other, that the difficulties in providing employment for convalescents from mental disease is far in excess of those connected with any other form of redemption work. People more willingly employ the criminal discharged from prison than a cured lunatic. The need of the help granted by the association is intense, and the form of help most beneficial is that which enables the patient to recover his strength in a convalescent home and then gives him work. As one speaker remarked, "It is enough to drive anyone mad again to be discharged from the asylum, where he has had every comfort, to face the world penniless, dependent on his own exertions, and yet to have the door of so many occupations shut in his face on account of the nature of his recent illness." During the last twelve months 7 cases passed 14 before the council, and the maintenance fund reached £561, a higher sum than it has ever done. The boarding out of convalescents in cottage homes in the country has been carried out with increasing success, and there is need of additional homes for this purpose. Higher rates are now paid per week for each boarder, and this has proved a wise expenditure. The council has decided to appoint local secretaries, and a number of ladies and gentlemen have signified their willingness to act as such. This will save considerably in postage and working expenses. More convenient offices have been secured in the Church House, and efforts are being made to make the work of the association more widely known and thus secure a larger number of subscribers. During the proceedings Dr. Mocatta promised a donation of £50 if a sum of £1,000 was raised by other benefactions. The Rev. H. Hawkins closed the meeting with a sketch of the origin and growth of the association, mentioning in the course of his remarks that the French society for the same object is far ahead of ours.

